# How are elite tennis matches won at Wimbledon? A comparison of close and one‐sided contests

**DOI:** 10.1002/ejsc.12063

**Published:** 2024-01-09

**Authors:** Anna Fitzpatrick, Joseph A. Stone, Simon Choppin, John Kelley

**Affiliations:** ^1^ School of Sport Exercise & Health Sciences Loughborough University Loughborough UK; ^2^ Sport and Physical Activity Research Centre Sheffield Hallam University Sheffield UK; ^3^ Sports Engineering Research Group Sheffield Hallam University Sheffield UK

**Keywords:** elite tennis, grass court tennis, match closeness, match statistics, successful performance

## Abstract

Notational analysis investigations of several sports have suggested that the performance characteristics associated with success differ by match closeness. It is not known whether this is the case in tennis. Therefore, this study aimed to first develop operational definitions for *closely contested* and *one‐sided* tennis matches, then establish whether the important performance characteristics in elite grass court tennis differ by match closeness. Data from 365 men and 374 women's Wimbledon single matches played between 2015 and 2017 were analyzed. Irrespective of match closeness, *points won of 0–4 shot rally length*, *first serve points won* and *baseline points won* were associated with winning matches, and *forced errors* and *unforced errors* were associated with losing matches, for both sexes. Spearman's rank‐order correlations demonstrated excellent agreement between the importance of the performance characteristics in *closely contested* and *one‐sided* men (*r*_*s*_ = 0.89, *p* < 0.001) and women's matches (*r*_*s*_ = 0.90, *p* < 0.001), respectively. Findings suggest that expected match closeness (of an upcoming match) should not necessarily influence decision‐making around practice design and match‐play strategy. Additionally, the operational definitions developed for *closely contested* and *one‐sided* matches developed here could be used in future studies to investigate different competitive contexts.

## Highlights

- This is the first study to develop clear operational definitions for *closely contested* and *one‐sided* tennis matches; these can be utilized in future research.
- In contrast to previous investigations of team sports, the most important performance characteristics for winning matches at Wimbledon did not differ between *closely contested* and *one‐sided* matches.
- Irrespective of match closeness, winning short points, first serve points and baseline points were most closely associated with winning elite, grass court tennis matches for men and women.
- Findings suggest that expected match closeness should not necessarily influence coaches and players' decision‐making around practice design or match strategy planning.

## 1 INTRODUCTION

Performance analysis is an objective method of recording and interpreting sports performance that allows valid and consistent quantification of key events (Baca, 2014). In elite‐level sport, competitions are often analyzed to evaluate individual and team performances (Hughes et al., 2002); this allows assessment of strengths and areas for improvement (Sarmento et al., 2013), quantification of winner–loser differences (García et al., 2014; Meffert et al., 2018) and identification of performance characteristics closely associated with success (Grambow et al., 2020, 2021; Reid et al., 2010). In turn, results can inform training designs and tactical decision making prior to competition, by indicating which aspects of match‐play may be the most important for coaches to focus on with their athletes (Csataljay et al., 2009; Grambow et al., 2021).

With a wide range of applications for athletes and coaches, performance analysis has become a valued discipline in many sports, but historically, its progress within tennis was limited (Martin et al., 2012). Over the last decade, however, researchers and practitioners have begun to address this issue. Contemporary studies have investigated aspects including sex‐based differences in players' stroke and movement dynamics at the Australian Open (Reid et al., 2016), court surface differences between match‐play characteristics at Roland Garros and Wimbledon (Fitzpatrick et al., 2019b) and comparisons between the match‐play demands of junior and professional tennis (Kovalchik et al., 2017). Additionally, a new data analysis technique, designed to enhance coaches' understanding of performance data, has been validated (Fitzpatrick et al., 2019a), and researchers have begun to explore the characteristics of doubles match‐play (Kocib et al., 2020; Martínez‐Gallego et al., 2020).

Fitzpatrick et al. (2019b) recently highlighted the importance of *points won of 0–4 shot rally length* (i.e., short points) in elite grass court tennis, revealing that male and female players who won more short points than their opponent won the match in 92% and 87% of cases, respectively. This can be somewhat explained by the high prevalence of short points, with 72% (for men) and 66% (for women) of all points ending within the first four shots on grass courts (Fitzpatrick et al., 2021). *First serve points won, first serve‐return points won* and *baseline points won* were also closely associated with success for players of both sexes (Fitzpatrick et al., 2019b). Based on these results, the authors advised that tennis coaches should afford sufficient practice time to serve, serve‐returns and point‐ending strategies when preparing their players for the grass court season. While the study provided useful information, additional context around Fitzpatrick et al.’s (Fitzpatrick et al., 2019b) findings would allow coaches to ensure specificity in their practical application.

According to Csataljay et al. (2009), identifying the performance characteristics closely associated with success is most pertinent for sporting contests in which the difference between winning and losing players is small. In this context, research from sports including basketball (García et al., 2014; Gomez et al., 2008), rugby union (Vaz et al., 2011), Gaelic football (Allister et al., 2018) and handball (Oliveira et al., 2012) has suggested that the performance characteristics associated with success may differ in *closely contested* matches compared to *one‐sided* matches. For example, Gomez et al. (2008) revealed that in *closely contested* basketball games (those with a final score difference between the two teams of 12 points or fewer), *defensive rebounds* best discriminated winning and losing teams, whereas in *one‐sided* games (those with a final score difference of more than 12 points) *assists* best discriminated winning and losing teams. Hence, Gomez et al. (2008) were able to recommend greater specificity within training sessions around offensive strategies and the technical actions that lead to field‐goal attempts.

In tennis, match closeness has primarily been considered from an economics perspective (Du Bois et al., 2007). Research in this area has suggested that the competitive balance of professional tennis matches (i.e., whether they are closely contested or not) influences the level of public interest; generally, people prefer to watch matches with uncertain or less predictable outcomes (Du Bois et al., 2007). Evidence from team sport research proposes match closeness as a potentially useful stratification category in notational analysis studies, for several reasons. Investigating the influence of match closeness on the importance of performance characteristics can not only improve the specificity of training but also inform match‐play strategy (Hughes et al., 2017). If, for example, *double faults* were shown to discriminate winning and losing players in *closely contested* tennis matches, but not in *one‐sided* matches, players may choose to adapt their serving strategy accordingly. Such investigations can also aid our understanding of sporting performance within different competitive contexts (Gómez et al., 2010). For example, tennis players often lose confidence in their second serve during close matches, leading to anxiety and a decline in their performance level (Rutherford, 2017). If players understood that hitting a double fault was unlikely to affect their likelihood of winning the match, their loss in confidence may be ameliorated, their anxiety reduced and their performance level maintained as a result. Furthermore, within their comprehensive investigation of hard court tennis match‐play, Reid et al. (2016) recommended that future studies examine effects of match closeness on match‐play performance characteristics. As such, analysis from the perspective of match closeness may provide further insight into important performance characteristics in elite grass court tennis, better inform match‐play strategies and enhance the specificity and context of associated practical applications. However, to date, no published research has investigated the characteristics of tennis match‐play from a match closeness perspective, so no operational definitions are available for ‘closely contested’ and ‘one‐sided’ matches.

Therefore, the aims of this study were (i) to develop clear operational definitions for *closely contested* and *one‐sided* tennis matches, based on expert coaches' assessments, and (ii) to establish whether the important performance characteristics of elite men and women's grass court tennis differ by match closeness (i.e., between *closely contested* and *one‐sided* matches).

## 2 METHOD

### 2.1 Matches

With institutional ethics approval, performance characteristics for men and women's Wimbledon singles matches contested between 2015 and 2017 (men: *n* = 381, women: *n* = 381) were obtained from the IBM Wimbledon Information System (IBM, 2019). Access to the data was provided by IBM, with permission granted by The All England Lawn Tennis Club. Data from incomplete matches (i.e., those involving retirements, walkovers or defaults) were excluded; 16 men's matches and 7 women's matches were excluded accordingly. The length of all matches in the sample (in terms of number of sets) is included as Supporting Information S1.

### 2.2 Match closeness

Five Lawn Tennis Association (LTA) Performance Coaches (*n* = 3 females, *n* = 2 males; age 41.8 ± 11.0 years, coaching experience 18.2 ± 10.3 years) took part in the study. All coaches were of British nationality and held LTA Level 4 Senior Performance Coach (*n* = 2) or Level 5 Master Performance Coach (*n* = 3) qualifications. Each coach had at least 8 years of coaching experience and all had previously competed as professional tennis players. Performance coaches with playing backgrounds were consulted specifically, due to their invaluable, experiential knowledge of the sport (i.e., knowledge gained from years of competing, developing in and/or coaching elite tennis (Woods et al., 2021)), and how this can enrich our understanding of elite sport and enhance associated empirical research (Greenwood et al., 2012).

The coaches were each given a Microsoft Excel (Microsoft Corp, Redmond, WA, USA) spreadsheet containing the final scores of all completed men and women's Wimbledon matches contested between 2015 and 2017. All data were anonymized, and the order of matches was randomized to reduce bias. The coaches were asked to independently assess the match scores and decide whether each match was *closely contested* or *one‐sided*, based on their experiential knowledge of the sport. The coaches' independent assessments agreed with each other in 91.9% of the matches (679 of 739). The coaches were then asked to confer and discuss 60 matches, whereby their independent assessments were inconsistent to reach a consensus on whether each was *closely contested* or *one‐sided*.

To determine objective operational definitions for *closely contested* and *one‐sided* matches in tennis, an explorative technique was used alongside the coaches' assessment. Different criteria—threshold values to maximize agreement—were tested for six statistics linked to match score (total number of points in the match, total number of sets in the match, mean number of games per set, mean number of points per set, percentage of games won by the losing player and percentage of points won by the losing player) to identify which criterion demonstrated the strongest agreement with the coaches' assessment. A criterion based on the *percentage of games won by the losing player* demonstrated the strongest agreement with the coaches' assessment for both sexes (see Table 1, ordered by sex and level of agreement). Dual combinations of the six statistics were also tested, but all demonstrated lower agreement with the coaches' assessment than the criteria based on the *percentage of games won by the losing player*.

**TABLE 1:** The number (and percentage) of matches whereby each tested criterion for closely contested matches agreed with the coaches' assessment.

Sex	*Closely contested* match criterion	Agreement with coaches' assessment
Men	Total number of sets >3	281/365 (77.0%)
	Mean number of points per set >57	287/365 (78.6%)
	Mean number of games per set >9	297/365 (81.4%)
	Total number of points >190	326/365 (89.3%)
	Percentage of points won by losing player >44%	331/365 (90.7%)
	Percentage of games won by losing player >38%	356/365 (97.5%)
Women	Total number of sets >2	257/374 (68.7%)
	Mean number of points per set >57	290/374 (77.5%)
	Mean number of games per set >8.7	304/374 (81.3%)
	Percentage of points won by losing player >43%	333/374 (89.0%)
	Total number of points >120	334/374 (89.3%)
	Percentage of games won by losing player >36%	358/374 (95.7%)

Agreeing with the coaches' assessment in 97.5% of men's matches (356 of 365) and 95.7% of women's matches (358 of 374), the criteria displayed in Table 2 were selected for the study.

**TABLE 2:** Operational definitions for one‐sided matches and closely contested matches for men and women.

Sex	Operational definition	
	*One‐sided match*	*Closely contested match*
Men	A match in which the losing player won ≤38.0% of games	A match in which the losing player won >38.0% of games
Women	A match in which the losing player won ≤36.0% of games	A match in which the losing player won >36.0% of games

Based on these operational definitions, each match in the dataset was stratified into one of the two groups: *closely contested* matches or *one‐sided* matches. Accordingly, 248 men's matches and 217 women's matches were classified as *closely contested*, with 117 men's matches and 157 women's matches classified as *one‐sided*.

### 2.3 Performance characteristics

The following commonly used performance characteristics, derived from O’Donoghue and Ingram (O'Donoghue et al., 2001) and O’Donoghue (O’Donoghue, 2005), were obtained for the winning and losing player in each match: number of aces, number of double faults, number of first serves attempted, number of first serves in, number of serve‐volley points played, number of serve‐volley points won, number of first serve points won, number of second serve points won, number of first serve‐return points won, number of second serve‐return points won, number of baseline points won, number of net points won, number of forehand and backhand winners, number of forehand and backhand forced errors, number of forehand and backhand unforced errors, total number of points played and total number of points won. Additionally, the number of points played and won of 0–4, 5–8 and 9+ shot rally length, respectively, and mean first serve speed were obtained for matches where a serve speed radar was available.

### 2.4 Reliability testing

Wimbledon's data entry team recorded the data (i.e., the data were secondary), so reliability testing was necessary (Verma, 2012). Following error detection and data cleaning, video recordings of four matches (two men's matches and two women's matches) were observed and coded independently by the principal researcher, using a NacSport (NacSport Elite, Las Palmas de Gran Canaria, Spain) custom‐notational analysis system. Inter‐rater reliability was calculated using Cohen's kappa coefficient, based on the analysis of 496 match‐play points, comparing the lead researcher's results with those recorded by Wimbledon's data entry team. Cohen's kappa coefficient was *k* = 0.99, which is defined as excellent (Fleiss, 1981).

### 2.5 Data processing and analysis

Data were stratified by sex, year, match outcome (i.e., winning player or losing player) and match closeness (i.e., *closely contested* or *one‐sided*), then normalized using the equations presented in Table A1 before being reduced to mean values (±*sd*).

For each performance characteristic, the winning player's performance was compared to that of their opponent (i.e., the losing player), to establish which player outscored the other. Then, the *Percentage of matches in which the Winner Outscored the Loser* (PWOL) was calculated for each performance characteristic (see Fitzpatrick et al. (2019a)) for a validation of the PWOL method). The PWOL method was introduced as a more practitioner‐friendly alternative to complex data analysis methods, such as point‐biserial correlations and *t* tests, to support coaches' understanding and interpretation of tennis match‐play data (Fitzpatrick et al., 2021). The PWOL method produces a value between 0% and 100%, which can be interpreted to indicate the importance of any performance characteristic in terms of winning matches. A PWOL of 50% for a particular performance characteristic indicates no association with success. As PWOL increases toward 100%, this indicates a stronger positive association with success, whereas as a PWOL close to 0% demonstrates a strong negative association with success (i.e., a strong association with losing matches) (Fitzpatrick et al., 2019a). PWOLs were therefore used in this study to indicate the relative importance of each characteristic in *closely contested* and *one‐sided* matches, for male and female players, respectively. Table B1 shows the full interpretation of PWOL values (Fitzpatrick et al., 2021).

To assess the agreement between the importance of each performance characteristic (i.e., their PWOLs) in *closely contested* matches and *one‐sided* matches, a Spearman's rank‐order correlation coefficient was calculated for each sex.

## 3 RESULTS

### 3.1 Men's results

**FIGURE 1:**
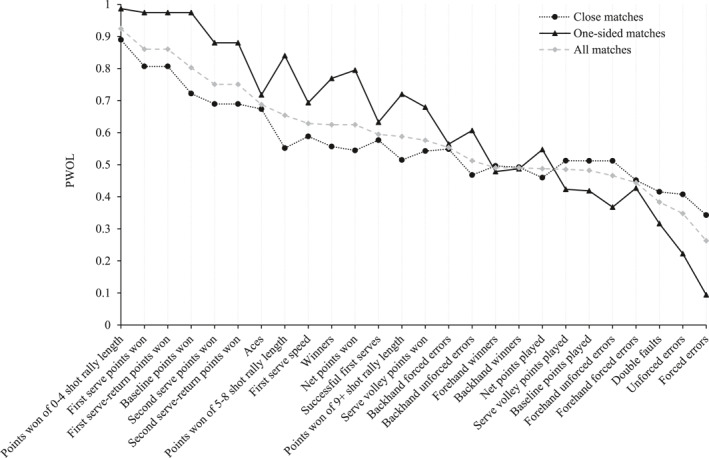
Men's PWOLs for each performance characteristic, for closely contested matches, one‐sided matches and all matches.

Figure 1 displays PWOLs for all performance characteristics in men's *closely contested* and *one‐sided* matches played between 2015 and 2017 at Wimbledon. Overall PWOLs for performance characteristics (i.e., PWOLs in *all* matches combined) are also displayed for context. Performance characteristics are displayed in descending order of PWOL for *all* matches (i.e., overall PWOLs; from left to right).

Figure 1 shows that, for men, *points won of 0*–*4 shot rally length*, *first serve points won*, *first serve‐return points won* and *baseline points won* demonstrated the highest PWOLs in both *closely contested* and *one‐sided* matches. *Forced errors*, *unforced errors* and *double faults* demonstrated the lowest PWOLs, irrespective of match closeness. Figure 1 also shows that *one‐sided* matches typically elicited a PWOL further from 50% than *closely contested* matches, for any given performance characteristic. In other words, most performance characteristics were more closely associated with match outcome in *one‐sided* matches than *closely contested* matches. Additionally, the difference in PWOLs between *closely contested* and *one‐sided* matches was generally smaller for characteristics with overall PWOLs (i.e., gray data points) closer to 50% and larger for those with overall PWOLs further from 50%. However, the difference between PWOLs in *closely contested* and *one‐sided* matches was also smaller (relatively) for specific performance characteristics including *aces*, *first serve speed* and *successful first serves*, and larger for characteristics including *forced errors* and *points won of 5–8 shot rally length*.

Spearman's rank‐order correlation coefficient, assessing the agreement between men's PWOLs for *closely contested* and *one‐sided* matches, was calculated as *r*_*s*_ = 0.89 (*p* < 0.001), demonstrating an excellent agreement (Hahs‐Vaughn et al., 2012).

### 3.2 Women's results

**FIGURE 2:**
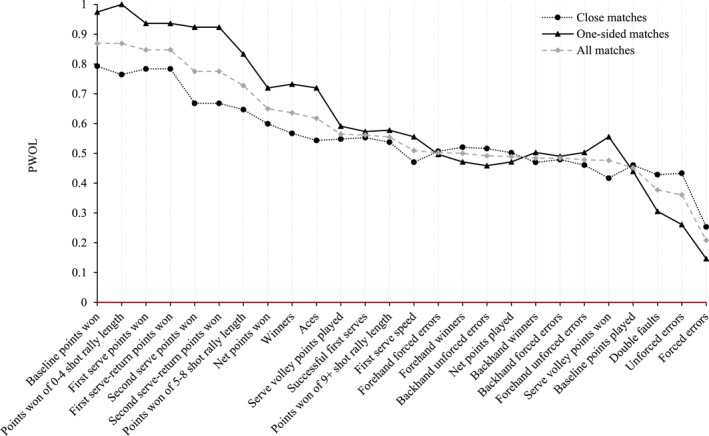
Women's PWOLs for each performance characteristic, for closely contested matches, one‐sided matches and all matches.

Figure 2 displays PWOLs for all performance characteristics in women's *closely contested* and *one‐sided* matches played between 2015 and 2017 at Wimbledon. Overall PWOLs for performance characteristics (i.e., PWOLs in *all* matches combined) are also displayed for context. Performance characteristics are displayed in descending order of PWOL for *all* matches (i.e., overall PWOLs; from left to right).

Figure 2 shows that, for women, *baseline points won, points won of 0–4 shot rally length*, *first serve points won* and *first serve‐return points won* demonstrated the highest PWOLs in *closely contested* and *one‐sided* matches. *Forced errors* and *unforced errors* demonstrated the lowest PWOLs, irrespective of match closeness. For women, as for men, *one‐sided* matches typically elicited a PWOL further from 50% than *closely contested* matches, for any given performance characteristic. Also reflecting men's results, the difference between women's PWOLs in *closely contested* and *one‐sided* matches tended to be smaller for those performance characteristics with overall PWOLs (i.e., gray data points) closer to 50% and larger for those with overall PWOLs further from 50%.

Spearman's rank‐order correlation coefficient, assessing the agreement between women's PWOLs for *closely contested* and *one‐sided* matches, was calculated as *r*_*s*_ = 0.90 (*p* < 0.001), demonstrating excellent agreement (Hahs‐Vaughn et al., 2012).

## 4 DISCUSSION

The aims of this study were to develop operational definitions for *closely contested* and *one‐sided* tennis matches, and to establish whether the important performance characteristics in elite men and women's grass court tennis differ by match closeness. During the process to define *closely contested* and *one‐sided* matches, it became apparent that the criteria for each differed by sex. As such, based on the experiential knowledge of five expert coaches, *closely contested* matches were defined as “matches in which the losing player won >38.0% of games (for men) or >36.0% of games (for women)”, and *one‐sided* matches were “matches in which the losing player won ≤38.0% of games (for men) or ≤36.0% of games (for women)”. Notably, no published operational definitions were available for *closely contested* and *one‐sided* matches in tennis.

Applying the operational definitions developed here, subsequent analysis revealed that for both sexes, the same performance characteristics exhibited the highest and lowest PWOLs, respectively, in *closely contested* and *one‐sided* matches. This observation was supported by Spearman's rank‐order correlations, which demonstrated excellent agreement between the PWOLs in *closely contested* matches and those in *one‐sided* matches. This shows that the order of importance of the performance characteristics (in terms of winning) was similar irrespective of match closeness; the performance characteristics that were the most (and least) important in *one‐sided* matches were also the most (and least) important in *closely contested* matches. These results are in contrast with those from several team sport investigations, which have suggested that the performance characteristics most closely associated with success differ in *closely contested* matches compared to *one‐sided* matches (Allister et al., 2018; García et al., 2014; Gomez et al., 2008; Oliveira et al., 2012; Vaz et al., 2011).

For players of both sexes, *points won of 0–4 shot rally length*, *first serve points won*, *first serve‐return points won* and *baseline points won* consistently exhibited the highest PWOLs. In men and women's *one‐sided* matches, PWOLs for these four characteristics indicated very strong associations with winning. In men's *closely contested* matches, *points won of 0–4 shot rally length*, *first serve points won* and *first serve‐return points won* exhibited PWOLs above 80%, demonstrating strong associations with winning. This shows that these three characteristics differentiate between winning and losing players, even in *closely contested* matches, and highlights their critical nature at Wimbledon. While the high prevalence of both short points and points won on serve partly explain this, these results are particularly pertinent for coaches of male players, who should be aware that dominance in these areas can greatly improve players' chances of winning, irrespective of match closeness. Additionally, O’Shannessy (O’Shannessy, 2019a) recently suggested that elite players spend approximately 90% of their practice time engaging in long, baseline rallies. In this context, the high PWOLs for *points won of 0–4 shot rally length* support the assertions of tennis practitioners that the importance of short points should not be underestimated within players' training sessions (Anderson, 2018; O’Shannessy, 2019b; Pretorius et al., 2019).

Although Spearman's correlations demonstrated excellent agreement between PWOLs in *closely contested* and *one‐sided* matches, it should be noted that this agreement pertains only to the rank‐order of the PWOLs, and not the PWOLs themselves. For men and women, *one‐sided* matches elicited PWOLs further from 50% than *closely contested* matches for most performance characteristics. Hence, the overall range in PWOLs was greater for *one‐sided* matches than for *closely contested* matches. In terms of PWOL interpretation, this means that each performance characteristic's association with match outcome (whether positive or negative) was typically stronger in *one‐sided* matches than in *closely contested* matches. Thus, the performance characteristics that were important in terms of winning in *closely contested* matches were even more important in *one‐sided* matches. Given the nature of the scoring system in tennis, this can be expected. Several performance characteristics shown to be associated with winning matches (i.e., those with PWOLs above 60%) pertain to the points won (e.g., *points won of 0–4 shot rally length*, *points won of 5–‐8 shot rally length*, *first serve points won*, *second serve points won*, *baseline points won* and *net points won*). The nature of the scoring system means that winning players intrinsically win considerably more points than losing players in *one‐sided* matches (Wright et al., 2013), whereas this is not necessarily the case in *closely contested* matches. Therefore, the likelihood is that in *one‐sided* matches, winning players will outperform losing players on the performance characteristics that pertain to the points won more often than in *closely contested* matches, leading to the comparatively higher PWOLs in *one‐sided* matches found here.

Interestingly, three of the men's serve‐related characteristics—*first serve speed*, *successful first serves* and *aces*—demonstrated relatively small differences in PWOLs between *closely contested* and *one‐sided* matches; contrastingly, characteristics such as *forced errors* and *points won of 5–8 shot rally length* demonstrated relatively greater differences. This suggests that, irrespective of whether these characteristics were associated with the match outcome, *first serve speed*, *successful first serves* and *aces* are not differentiating factors of match closeness in men's grass court tennis. In contrast, performance characteristics that exhibited greater differences in PWOLs between *closely contested* and *one‐sided* matches, such as *forced errors* and *points won of 5–8 shot rally length*, do appear to differentiate between *closely contested* and *one‐sided* men's matches.

With respect to the limitations, it is worth noting that all coaches involved in this research were British; coaches of other nationalities may have different perspectives on which score‐lines are indicative of *closely contested* or *one‐sided* matches. Additionally, the method used to develop operational definitions in this study was retrospective, based solely on final match score, which may have limitations, as mid‐match momentum swings could not be considered. Therefore, the method could be further developed by incorporating an element of live scoring, whereby coaches classify the match as close or one‐sided as the match progresses.

There are several practical applications of this study. From a research perspective, the operational definitions developed here could be used in the future to facilitate analyses of different competitive contexts (e.g., match‐play on other court surfaces or elite junior competitions). Current findings also suggest that future studies investigating important performance characteristics in grass court tennis need not stratify their data by match closeness or account for any associated effect.

In terms of coaching applications, guided by Pinder's (Pinder et al., 2011) representative learning design framework, the consistent importance of *points won of 0–4 shot rally length* revealed here suggests that coaches should ensure short rallies and point‐ending strategies are fundamental aspects of players' grass court training. The results also indicate that expected match closeness (e.g., of an upcoming match) should not necessarily influence coaches' decision‐making around the design of players' training sessions or match strategy planning. However, this interpretation pertains specifically to the practical design of training sessions, rather than any psychological preparation that players might undertake prior to a potentially tough match (Bollettieri, 2015; Hoskins, 2003). Consider, for example, a lower ranked player preparing to face a considerably higher ranked opponent (and therefore expected to lose comfortably). The lower‐ranked player may psychologically plan to simply ‘stay in each point’ for as long as possible, to give themselves a chance. Our results indicate that this strategy alone is unlikely to turn an expected loss into a win. Rather, this approach could be employed at specific times during a match (e.g., when the opponent has the advantage in a rally), but should crucially be underpinned by an aggressive mentality and plan to implement point‐ending strategies often within the first four shots.

This study investigated *closely contested* and *one‐sided* tennis matches played on grass courts; future research analyzing differences between court surfaces is warranted. The current dataset comprised 3 years of Wimbledon singles match‐play, grouped to increase men and women's sample sizes. Analysis of longitudinal data stratified by year would reveal changes over time, from the perspective of match closeness, and research exploring “big data” (e.g., Hawk‐Eye ball‐ and player‐tracking coordinates) may provide further insight into differences between *closely contested* and *one‐sided* matches. Furthermore, a potential follow‐up the study could aim to validate the operational definitions developed here by asking coaches of different nationalities to reflect on them. Finally, it is common (and encouraged) for operational definitions in performance analysis to be re‐evaluated and evolve over time (Williams, 2012). Thus, these operational definitions could be further developed and applied to investigate whether there are ‘critical points’ within *closely contested* matches that determine the outcome of the match.

## 5 CONCLUSION

This study was the first to publish operational definitions for *closely contested* and *one‐sided* men and women's tennis matches. The definitions presented here could be adopted to investigate different competitive contexts in future, and further developed to incorporate live scoring and mid‐match momentum swings. The definitions were applied to elite men and women's grass court matches to investigate whether the importance of each performance characteristic differed by match closeness. Spearman's rank‐order correlations revealed excellent agreement between the PWOLs in *one‐sided* matches and *closely contested* matches, for both sexes. Therefore, there is no need for researchers to stratify performance data by match closeness in future investigations of elite grass court match‐play, or for coaches to alter players' training sessions accordingly. Finally, although the *order* of importance of the performance characteristics did not differ, it should be noted that the *level* of importance of several characteristics was greater in *one‐sided* matches than in *closely contested* matches. Coaches should consider if and how this might guide players' match‐play preparation.

## CONFLICT OF INTEREST STATEMENT

No potential conflict of interest was reported by the authors.

## Supporting information

Supporting Information S1

## APPENDIX A

**TABLE A1:** Normalized performance characteristic equations derived from O’Donoghue and Ingram (Greenwood et al., 2012) and O’Donoghue (O'Donoghue et al., 2001).

Performance characteristic	Equation
Aces (%)	Number of aces/number of serves performed × 100
Double faults (%)	Number of double faults/number of points served × 100
Successful first serves (%)	Number of first serves in/number of first serves attempted × 100
First serve points won (%)	Number of first serve points won/number of first serve points played × 100
First serve‐return points won (%)	Number of first serve‐return points won/number of first serve‐return points played × 100
Second serve points won (%)	Number of second serve points won/number of second serve points played × 100
Second serve‐return points won (%)	Number of second serve‐return points won/number of second serve‐return points played × 100
Serve‐volley points played (%)	Number of serve‐volley points played/number of serve points played × 100
Serve‐volley points won (%)	Number of serve‐volley points won/number of serve‐volley points played × 100
Net points played (%)	Number of net points played/number of rally points played × 100
Net points won (%)	Number of net points won/number of net points played × 100
Baseline points played (%)	Number of baseline points played/number of rally points played × 100
Baseline points won (%)	Number of baseline points won/number of baseline points played × 100
Winners (%)	Number of winners/number of rally points played × 100
Forced errors (%)	Number of forced errors/number of rally points played × 100
Unforced errors (%)	Number of unforced errors/number of rally points played × 100
Forehand winners (%)^a^	Number of forehand winners/total number of groundstroke winners × 100
Backhand winners (%)^a^	Number of backhand winners/total number of groundstroke winners × 100
Points won of 0–4 shot rally length (%)	Number of points won of 0–4 rally length/number of points played of 0–4 rally length × 100
Points won of 5–8 shot rally length (%)	Number of points won of 5–8 rally length/number of points played of 5–8 rally length × 100
Points won of 9+ shot rally length (%)	Number of points won of 9+ rally length/number of points played of 9+ rally length × 100

^a^ Equations for forehand/backhand forced errors and forehand/backhand unforced errors replicate those for forehand/backhand winners.

## APPENDIX B

**TABLE B1:** PWOL interpretation.

PWOL (%)	Interpretation
0% ≤ PWOL < 20%	Strong association with losing
20% ≤ PWOL < 40%	Association with losing
40% ≤ PWOL < 60%	No association with match outcome
60% ≤ PWOL < 80%	Association with winning
80% ≤ PWOL ≤ 100%	Strong association with winning
