# Supplementary material for: How are elite tennis matches won at Wimbledon? A comparison of close and one‐sided contests
Source: Eur J Sport Sci. 2024 Jan 9;24(2):190–9. doi: 10.1002/ejsc.12063 (PMC11235650; doi:10.1002/ejsc.12063)
Supplement: Supplementary file 1 — Supporting Information S1 [file EJSC-24-190-s001.docx]

**Supplementary Information**

**Table 1**. Number of matches of each length (in terms of set) per year, contested by men and women.

| **Match length** | **Men** | | | **Women** | | |
| --- | --- | --- | --- | --- | --- | --- |
|  | **2015** | **2016** | **2017** | **2015** | **2016** | **2017** |
| **2 sets** |  |  |  | 86 | 88 | 82 |
| **3 sets** | 59 | 60 | 59 | 39 | 36 | 43 |
| **4 sets** | 34 | 37 | 37 |  |  |  |
| **5 sets** | 31 | 27 | 21 |  |  |  |
| **Total** | 124 | 124 | 117 | 125 | 124 | 125 |
